# *Allium formosum* Sennikov & Lazkov (Amaryllidaceae), a new species from Kyrgyzstan

**DOI:** 10.3897/phytokeys.21.4130

**Published:** 2013-04-02

**Authors:** Alexander N. Sennikov, Georgy A. Lazkov

**Affiliations:** 1 Botanical Museum, Finnish Museum of Natural History, P.O. Box 7, 00014 University of Helsinki, Finland; & Herbarium, Komarov Botanical Institute of Russian Academy of Sciences, Prof. Popov str. 2, 197376 St. Petersburg, Russia; 2 Laboratory of Flora, Institute of Biology and Soil Science, Kyrgyz Academy of Sciences, 720071 Bishkek, Kyrgyzstan

**Keywords:** Central Asia, conservation, endemism, hotspot, new species, plant protection, Western Tian-Shan

## Abstract

*Allium formosum* Sennikov & Lazkov **sp. nov.** is described as new to science and illustrated. This species is the second member of *Allium* sect. *Spathulata* F.O.Khass. & R.M.Fritsch, being different from *Allium spathulatum* F.O.Khass. & R.M.Fritsch in larger, broader, obtuse and more intensely purple-coloured tepals, and in a more robust habit. It is a local endemic of Babash-Ata Mt. Range situated east of Fergana Valley in Kyrgyzstan, recommended for legal protection as Endangered because of the very small population size in its only locality.

## Introduction

The genus *Allium* L. is highly speciose in the former Soviet part of Central Asia. The latest synopsis (Khassanov 2008) lists nearly 250 species and subspecies, and this number is constantly growing with descriptions of new species from nearly all mountainous areas of this region.

Kyrgyzstan is a Central Asian country with a rich native flora, assessed at nearly 3800 native species of vascular plants (Lazkov and Sultanova 2011) and still remaining seriously underexplored (Kamelin 2002). The old *Flora of Kirghiz SSR* (Nikitina and Kaschenko 1951) listed 67 species of *Allium*; this number has been significantly increased with a further exploration of the country by local botanists and *Allium* taxonomists. The current inventory is being performed after publication of the new checklist of vascular plants of Kyrgyzstan (Lazkov and Sultanova 2011) that counted 85 species of *Allium*, making an increase of 25% for the last 60 years.

During the years 2009–2011 botanists of the Botanical Museum of the University of Helsinki, in collaboration with the Institute of Biology and Soil Science of the Kyrgyz Academy of Sciences, made expeditions to collect plants in Kyrgyzstan. The expeditions focused on the most difficult and diverse taxonomic groups, with emphasis on local endemics. Several taxonomic novelties and new records resulted from these travels (Sennikov 2010, 2011; Sennikov et al. 2011). One of the new species belongs to *Allium* sect. *Spathulata* F.O.Khass. & R.M.Fritsch, and its description is presented here.

*Allium* sect. *Spathulata* was established (Khassanov 2000) for the single species *Allium spathulatum* F.O.Khass. & R.M.Fritsch that was discovered only recently (Fritsch et al. 1998). This miniature plant has a very limited local occurrence and remained undetected in spite of its location within the limits of the famous Sary-Chelek Nature Reserve (Kyrgyzstan), where its type locality is situated nearby the headquarters of the Nature Reserve. These plants are so rare and minute that they were unnoticed for 100 years of botanical studies in the Nature Reserve.

When originally described, *Allium spathulatum* was considered unique in *Allium* subgen. *Allium* because of the presence of spathules and papyraceous tunics, ovoid bulbs, and the absence of bulblets (Khassanov 2000). Although this species was not included in the molecular phylogeny of *Allium* (Friesen et al. 2006) yet, which still embraces selected sections only, it was speculated to be less specialised in the subgenus from the primitive state of its several characters, e.g. an inflorescence with numerous long bracteoles (“spathules”), papery-like bulb tunics, and a symmetric karyotype with small satellites (Fritsch et al. 1998; Friesen et al. 2006).

## Materials and methods

A description of the new taxon and its relative is based on herbarium vouchers and field observations, following the standard used in the taxonomy of *Allium*. One population per species is used. Measures are taken from dried plants.

Distribution maps are compiled using R software environment for statistical computing and graphics (R Development Core Team 2008). The base maps are taken from the Digital Chart of the World, Arc/INFO resource provided by the Environmental Systems Research Institute, Inc., the Pennsylvania State University Libraries.

## Data resources

Specimen information is deposited in the database of records in vascular plants of Kyrgyzstan (Sennikov and Lazkov 2012) that is published also through the Global Biodiversity Information Facility (GBIF) and in the Dryad Data Repository at doi: 10.5061/dryad.pq87t

## Taxonomic treatment

### Allium formosum

Sennikov & Lazkov (Allium sect. Spathulata F.O.Khass. & R.M.Fritsch) sp. nov.

urn:lsid:ipni.org:names:77126319-1

http://species-id.net/wiki/Allium_formosum

Fig. 1

#### Latin

*Ab Allio spathulato statura majore (caulibus ad 30 cm, nec ad 20 cm altis), spathulis brevioribus paucioribus, floribus pluris (ad 30, nec ad 20), tepalis obscuriore roseolo-purpureis, longioribus (6–7.5 mm, nec 4–5.5 mm longis) latioribusque (2–2.5 mm, nec 2 mm latis), apice obtusioribus (nec acutis) basi subrotundis (nec distincte angustatis) differt*.

#### Type.

Kyrgyzstan. Babash-Ata Range: Kara-Köl River valley, left riverside, alt. 1650 m, 41.53°N, 72.68°E, 14.07.2010, *A. Sennikov & G. Lazkov* 132 (H 1750496, holotype; isotypes FRU, H 1750497).

#### Description.

Bulbs subglobose, 7–8 mm in diameter, ca. 8 mm long, inner tunices slightly violaceous, very thin, transparent, papyraceous, with several longitudinal nerves, outer ones light-grey, decomposing. Bulblets missing. Scape single, 20–25 (30) cm long, up to 1.5 mm in diameter, solid, dark green with a slight purple tint at the base. Leaves 2(3), linear, not exceeding the stems, upright, with the blade up to 20 cm long, ca. 1.5 mm wide, round-appressed and fistulose in the section, dark green, glabrous, with sheaths up to 10 cm long. Spathe membranous, completely divided into two elongate valves 4–6 mm long, reflexed. Inflorescence hemisphaerical, rather lax, with 7–30 developed flowers and ca. 5 abortive buds; pedicels thin, basally thickened, straight, dark-green, of the same length, ca. 1.5 cm long, some of them being embraced in narrow spathules ca. 1 mm long. Perianth cupuliform, intensively pinkish-purpureous in the upper two thirds, basally whitish, with dark-purpureous median veins. Tepals 6–7.5 mm long, 2–2.5 mm wide, oblong, obtuse at the apex, subrotund and only very slightly narrowed to the base. Filaments shorter than tepals, 2.5–3 mm long, white, connected and fused with sepals at the base, outer ones with the triangular base, inner ones broader, tricuspidate. Anthers ca. 0.4 mm long, yellow. Ovary ca. 2 mm long, 2–2.5 mm in diameter, subglobular. Style slightly over 1 mm long, white. Capsule and seeds not known.

**Figure 1.**
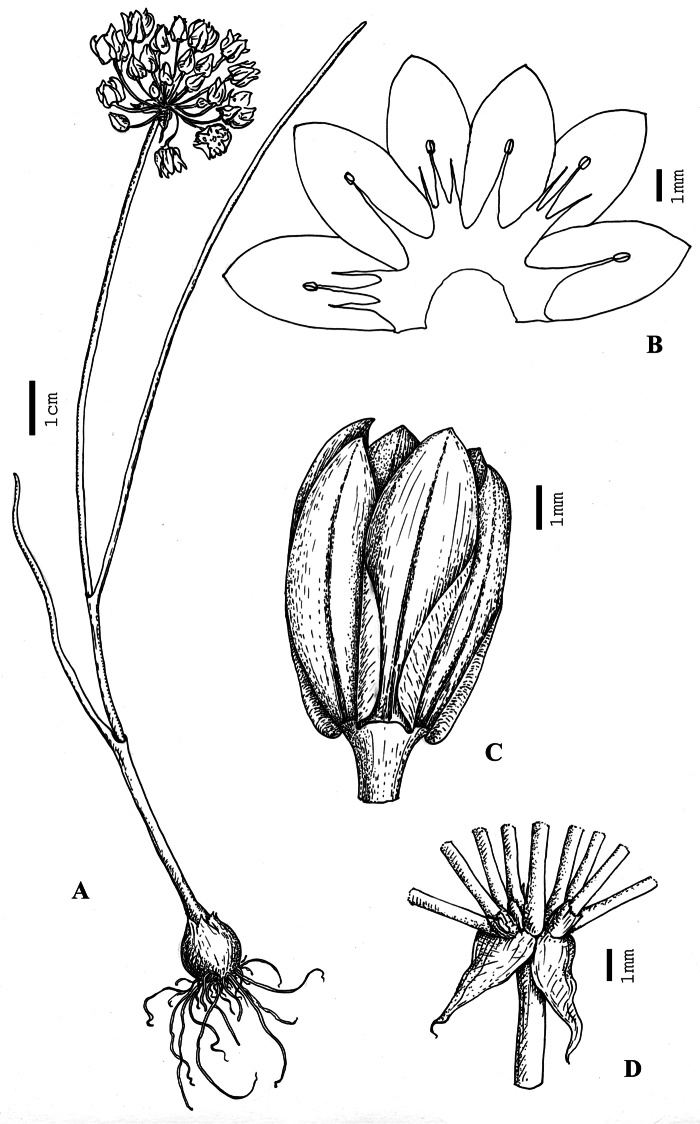
*Allium formosum*. **A** habit. **B** inner side of the perianth with stamina. **C** flower. **D** basal part of the umbella. Drawn from the type (H 1750496).

**Figure 2.**
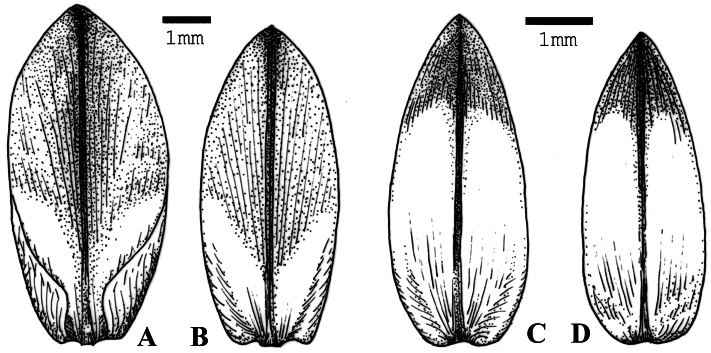
*Allium formosum*. **A** outer tepal **B** inner tepal. Drawn from the type (H 1750496). *Allium spathulatum* **C** outer tepal **D** inner tepal. Drawn from *Lazkov* s.n. (H 1750506).

#### Phenology.

Flowering in July, fruiting unknown.

#### Ecology.

The species occurs in the low-altitude forest zone (altitudes of ca. 1600–1700 m) in river valleys, on open sunny slopes with sparse savannoid vegetation, sheltered by stones. The plants grow clustered in small patches, suggesting the most successful establishment nearby mature plants (vegetative reproduction is not known in this section).

#### Distribution.

**Figure 3.**
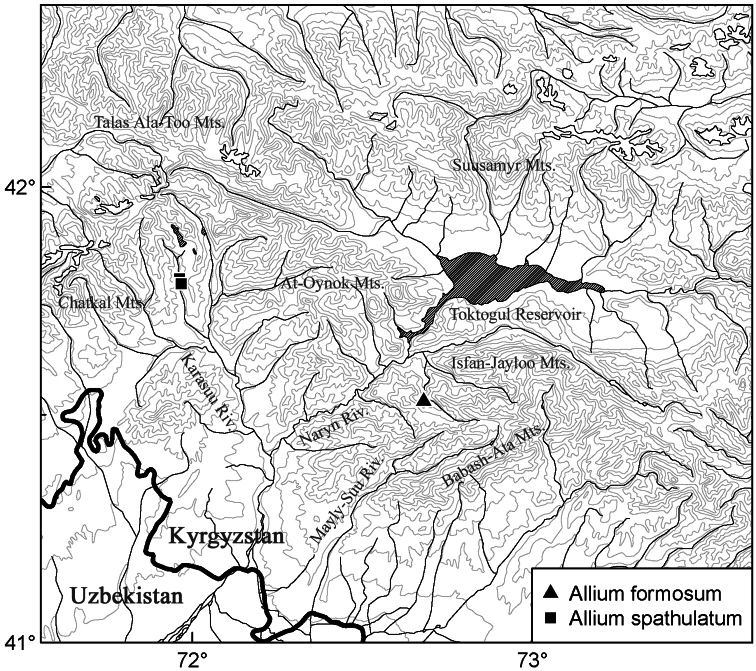
Distribution areas of *Allium formosum* and *Allium spathulatum*.

Possibly a narrow endemic of Babash-Ata Mt. Range, Kyrgyzstan (Fig. 3), so far known from the type locality only.

#### Proposed conservation status.

The distribution area of this species is like those of other local endemics of the mountains east of Fergana Valley. A single locality is known, where ca. 10 clusters of flowering plants were noticed. Even though no immediate threat was observed, the area is in active use, first of all for grazing and mining. For this reason and because of the very limited distribution area and a small size of the only population known to date (criterium D: population size estimated to number fewer than 250 mature individuals), this taxon may be recommended for protection as Endangered (IUCN 2001).

#### Etymology.

The new species is named because of its elegant habit and beautiful colouration of the perianth, transitional between deeply pink and purple; Lat. *formosum* = beautiful.

## Discussion

*Allium spathulatum* F.O.Khass. & R.M.Fritsch has been recently described from Chatkal Range, Sary-Chelek Nature Reserve, vicinities of Arkyt village (Fritsch et al. 1998). This species was recollected on 10.06.2010 by G. Lazkov from the slopes on the left side of Kojo-Ata River valley, situated immediately outside the entrance to Sary-Chelek Nature Reserve, in ca. 1 km from the original locality. The plants collected at that site (FRU, H 1750495, 1750506) were in a complete agreement with the protologue.

The plants from Babash-Ata Mts., Kara-Köl River differ from *Allium spathulatum* mostly in having longer and broader tepals, which are obtuse at the apex and less narrowed to the base (Fig. 2), and in a larger size of the whole plant. The flowers seem to be more numerous. Some pedicels are embraced by spathules which are generally less developed (shorter and less numerous) than in *Allium spathulatum*. These differences warrant the segregation of these plants into a separate taxon; the rank of species is preferred here because the differences are complex and constant. Another example of a similar distinction in the floral characters is the Central Asian pair *Allium tianschanicum* Rupr. – *Allium kokanicum* Regel, where major differences are also in the size and shape of tepals.

Another difference is observed in the shape of flowers which open less widely and thus look cupuliform in *Allium formosum* (campanulate in *Allium spathulatum*). However, this difference may appear dependent on weather conditions and needs to be proven by further observations.

The distance between the localities of *Allium spathulatum* and *Allium formosum*, both narrow endemics of mountains surrounding the eastern end of Fergana valley, is about 60 km (Fig. 3). The area of the eastern part of Chatkal Range and the northern outliers of Fergana Range (Babash-Ata and neighbouring mountains) is well known for the concentration of many narrow endemics, being a hotspot of plant diversity in Western Tian-Shan (Lazkov et al. 2002). The territories surrounding the eastern end of Fergana valley harbour many narrow endemics of *Allium* as well (see a brief review in Fritsch et al. 1998), and our discovery stresses the need of further explorations and plant protection in this area. Because of vulnerability of the species, we anticipate its inclusion in the forthcoming Red Data Book of Central Asia and the next edition of the Red Data Book of Kyrgyzstan.

The present state of the *Allium* research in Central Asia, especially descriptions of new species that are still regularly published, clearly show that the species inventory in this speciose and difficult genus is far from complete. In the absence of elaborated molecular phylogenies we feel premature to speculate on the origin and age of our newly discovered species.

### The members of *Allium* sect. *Spathulata* may be keyed out as follows

**Table:** 

1	Plants up to 20 cm tall. Tepals narrowly oblong, 4–5.5 mm long, 2 mm wide, acute at the apex and narrowed to the base, pinkish in the upper third	*Allium spathulatum*
–	Plants up to 30 cm tall. Tepals oblong, 6–7.5 mm long, 2–2.5 mm wide, obtuse at the apex, subrotund and very slightly narrowed to the base, intensely pinkish-purpureous in the upper two thirds	*Allium formosum*

### Other specimens examined

*Allium spathulatum* F.O.Khass. & R.M.Fritsch

Kyrgyzstan. Chatkal Range (S side): immediately S of Sary-Chelek Nature Reserve, left side of Kojo-Ata River valley, on rocks, 41.7° N, 71.9° E, 10.06.2010, *G. Lazkov* (FRU, H 1750495, 1750506).

## Supplementary Material

XML Treatment for Allium formosum
